# Q3: A Compact Device for Quick, High Precision qPCR

**DOI:** 10.3390/s18082583

**Published:** 2018-08-07

**Authors:** Marco Cereda, Alessandro Cocci, Davide Cucchi, Lillo Raia, Danilo Pirola, Lorenzo Bruno, Pietro Ferrari, Valentina Pavanati, Giorgia Calisti, Francesco Ferrara, Alessandro P. Bramanti, Marco A. Bianchessi

**Affiliations:** 1 STMicroelectronics S.r.l., I-20864 Agrate Brianza, Monza Brianza, Italy; marco.cereda@st.com (M.C.); alessandro.cocci@st.com (A.C.); davide.cucchi@st.com (D.C.); lillo.raia@st.com (L.R.); danilo.pirola@st.com (D.P.); lorenzo.bruno@st.com (L.B.); pietro.ferrari@st.com (P.F.); valentina.pavanati@st.com (V.P.); marco.bianchessi@st.com (M.A.B.); 2 CNR IMM—MDM, I-20864 Agrate Brianza, Monza Brianza, Italy; giorgia.calisti-ext@st.com; 3 STMicroelectronics S.r.l., I-73100 Lecce, Italy; francesco.ferrara@st.com

**Keywords:** Lab-on-a-Chip, qPCR, real-time PCR, Q3, point-of-care tests, low-cost devices, miniaturization, on-chip diagnosis

## Abstract

An accurate and easy-to-use Q3 system for on-chip quantitative real-time Polymerase Chain Reaction (qPCR) is hereby demonstrated, and described in detail. The qPCR reactions take place inside a single-use Lab-on-a-Chip with multiple wells, each with 5 to 15 µL capacity. The same chip hosts a printed metal heater coupled with a calibrated sensor, for rapid and accurate temperature control inside the reaction mixture. The rest of the system is non-disposable and encased in a 7 × 14 × 8.5 (height) cm plastic shell weighing 300 g. Included in the non-disposable part is a fluorescence read-out system featuring up to four channels and a self-contained control and data storage system, interfacing with an external user-friendly software suite. Hereby, we illustrate the engineering details of the Q3 system and benchmark it with seamlessly ported testing protocols, showing that Q3 equals the performance of standard commercial systems. Overall, to the best of our knowledge, this is one of the most mature general-purpose systems for on-chip qPCR currently available.

## 1. Introduction

Polymerase Chain Reaction (PCR) amplification of nucleic acids increases the concentration of target nucleotide sequences, when present, exploiting the peculiar characteristics of the macromolecule [1]. Whenever DNA or RNA must be searched for, starting from undetectable concentrations, PCR is the gold standard technique [2]. In its quantitative version (quantitative real-time PCR, or qPCR) it also provides an estimation of the initial concentration of target sequences, which is useful in many cases [3]. Examples include Human Immunodeficiency Virus (HIV) monitoring in seropositive patients, for therapy tuning [4], and quantification of alimentary pathogens in some foodstuff (such as seafood) where toxicity depends on the concentration [5].

The already huge importance of molecular diagnostics is bound to grow hand in hand with our knowledge of the genome; and the latter, in turn, is increasing at a fast pace. Thus, qPCR-based monitoring and diagnostics will be more and more pervasive within the medical and biological sciences. The real breakthrough, however, is going to take place when such tests become available outside the classical environments, like hospitals and specialized laboratories. A medical office, an ambulance, even a domestic environment are going to be the future settings for quick and/or frequent genomic analyses. This will require, however, Lab-on-a-Chip (LoC) implementations.

Not surprisingly, several such implementations have been attempted over the last two decades, the earliest dating back to the late 1990s [6] in which a number of different technological approaches and solutions have been tackled (for a critical review, see [7] and references therein). This also makes for the complexity of implementing this now universal laboratory technique in a miniaturized system.

In fact, PCR in itself is simple. The core idea is to denature double-stranded into single-stranded DNA sequences, then using each strand as template to create new, complementary strands out of nucleotides in solution—using specific starting sequences (primers) to ensure that this process takes place on the intended sequence only, if present. The iteration of these two steps leads the concentration to grow exponentially up to detectable and quantifiable levels. The systems implementing this type of process are conceptually simple. The crucial issue is the precision required by each subsystem, especially those for temperature control and detection. In fact, temperature controls the kinetics of primers’ annealing and polymerization in the cold phase (typically 55–60 °C), as well as the denaturing process in the hot phase (typically 95 °C), during which the two strands must separate preserving their integrity. In turn, the detection system (usually optical) must be accurate, especially with qPCR. All of this adds to the usual complexity in porting larger systems onto the chip scale. In addition, the reaction chambers must guarantee proper containment of the fluid, which, at microliter quantities, could quickly evaporate. Finally, a LoC system should be easily adaptable to any specific detection protocol, just as larger-scale instruments, simply changing the reagents and tuning the thermal cycling and the detection parameters.

State-of-the-art of qPCR LoC devices are mostly prototypal devices—that is, not optimized for industrialization. Generally, they do not undergo extensive validation on different targets, and in comparison with gold-standard qPCR instruments. Sometimes, they are not that portable either [7,8].

Here we show a complete and mature general-purpose LoC device for qPCR, developed by STMicroelectronics (ST). Denominated Q3-Plus V2 (Q3)—non-commercial name, from “Qualitative, Quantitative and Quick”—it is easy to use, portable, fast, and completely comparable with larger, standard qPCR instruments as to the performance. While a few PCR applications have been demonstrated with earlier versions of this instrument [9], in the present version it is mature on an industrial scale, thanks to extensive and fine-tuned engineering of both disposable and non-disposable parts and validation on standard benchmark. The instrument has also obtained the CE mark as laboratory instrumentation for research use.

## 2. Q3: System Description

According to a classification, Q3 performs time domain PCR, meaning that all the reactions and thermal cycling occur in the same chamber where the conditions change over time, without the need of moving the solution around the chip—with area saving. It is based on a classical implementation, including thermocycling and fluorescence-based optical read-out. Received protocols can then be ported, virtually seamlessly, from larger instruments, and even if some tweaking is likely possible to optimize them for Q3, their use “as are” is generally completely satisfying. This of course makes the development of Q3 applications quite smooth. Melting analysis of the PCR-amplified products is also possible on Q3, as well as performing isothermal DNA/RNA amplification and detection protocols like Loop-mediated isothermal Amplification (LAMP) [10]. The optical fluorescence system, together with part of the thermocycler—and, of course, the control system—are part of the non-disposable unit, the instrument, in its most recent version denominated Q3-Plus V2. The LoC cartridge, or disposable unit, includes the reaction wells and the other part of the thermocycler, as detailed below.

### 2.1. Non-Disposable Unit (Instrument)

**Figure 1:**
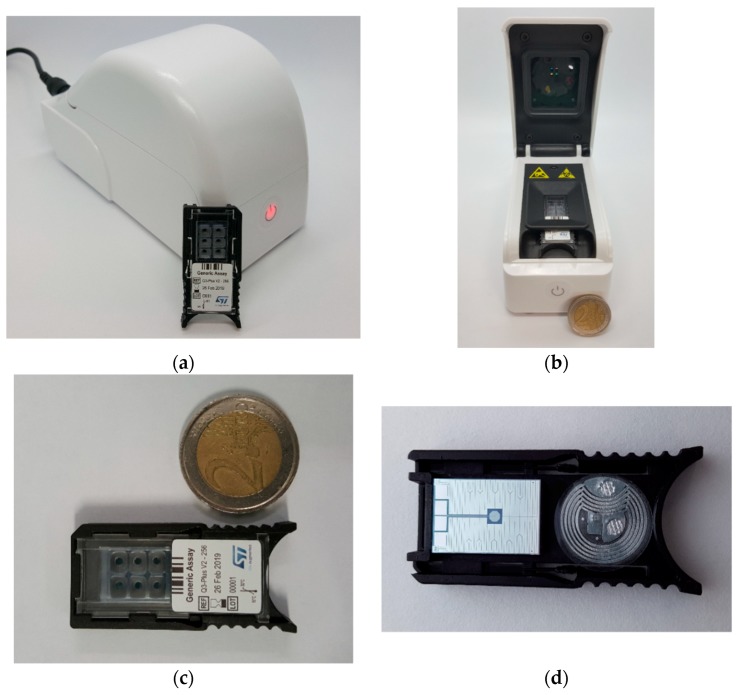
(**a**) The Q3-Plus V2 instrument closed, with the disposable unit (LoC cartridge) for comparison; (**b**) View of the open instrument. The optical system is inside the upper lid. In the lower part, the housing for the cartridge is visible, under which the control electronics and the cooling fan are positioned; (**c**) Front view of the cartridge, in which the six wells are visible; (**d**) Back view of the cartridge, evidencing the heater and temperature sensor on the flipside of the silicon die, as well as an RFID tag.

Overall, it is contained in a 7 × 14 × 8.5 (height) cm plastic shell and weighs only 300 g (Figure 1a,b). The upper lid, when open, unveils the embedded optical system (excitation sources, filters, and optical read-out sensors). The lower part of the instrument includes a sample holder apt to contain the LoC cartridge or disposable unit. The control electronics fits the space beneath the LoC cartridge, along with a cooling fan. Connection with a PC or smartphone/tablet is possible through a Universal Serial Bus (USB) port and Bluetooth^®^ connection, for programming as well as result read-out and storage. The Q3 instrument also possesses an embedded memory that stores all the data independently of the operating mode, as a backup; the analysis results can then be recovered from the instrument memory at any time.

### 2.2. Disposable Unit (LoC Cartridge)

The disposable unit is a 21.9 × 46.9 × 6.2 mm single-use LoC cartridge weighing less than 4 g. It includes reaction wells (six in this version; Figure 1c) carved into 3-mm thick, medical grade, transparent polycarbonate and covered with a poly(methyl methacrylate) sliding cap, for better insulation and to prevent contamination. The bottom of the wells is a 12.2 × 17.7 × 0.6 mm silicon die, which constitutes the core of the LoC cartridge, and undergoes a specific chemical treatment to ensure proper liquid confinement and optimal qPCR-compatibility. More in detail, the die surface is first covered with silicon dioxide, which is highly hydrophilic and qPCR-compatible; then a hydrophobic negative photoresist is deposited, which is subsequently patterned by photolithography and removed only from a circular area at the center of each reaction well—thus re-exposing the silicon dioxide there, where the reaction mixture will stay confined. The silicon die also hosts a heater and a temperature sensor on its flipside (details below; Figure 1d).

The wells can be preloaded at manufacturing with lyophilized reagents (qPCR reaction mixtures); alternatively, the cartridges can be provided without reagents, in which case the final user loads them in standard liquid format. The exact composition of the reaction mixtures will depend on the type of analysis—that is, on the targets—especially as to the primers and probes employed, while the physical structure of the cartridges is the same. Virtually any qPCR protocol developed for larger, standard qPCR instruments can be smoothly ported to Q3. In fact, some applications of Q3 have already been demonstrated [11,12,13] and several assays are currently under development.

The wells always come preloaded with wax, keeping the lyophilized reagents in place during chip storage, before the test is run. The prepared sample (purified DNA/RNA in aqueous solution, e.g., extracted from blood or other kinds of biological samples) must be pipetted into the wells to start an analysis—together with the reaction mixtures, if they are not already present in lyophilized form. The wells have either 5 or 15 µL capacity (total reaction volume, i.e., reaction mixture plus sample), depending on the cartridge version, to fit different protocol requirements. During the first heating ramp the wax will melt—between 45 and 55 °C, depending on the type—and raise to the top, where it will form a floating, liquid cap preventing evaporation of the mix and sample.

A Radio-Frequency Identification (RFID) tag on the cartridge flipside (Figure 1d) allows storing manufacturing information on the cartridge itself, as well as on the pre-loaded reagents—when present. Such information can then be read by the Q3-Plus V2 instrument, which can also write some new information inside the RFID tag—for instance, the results of the analysis run with that cartridge.

### 2.3. Thermal Control

Thermal cycling can be programmed depending on the protocol, and is tuned by a microcontroller through a Proportional-Integrative-Derivative (PID) algorithm.

**Figure 2:**
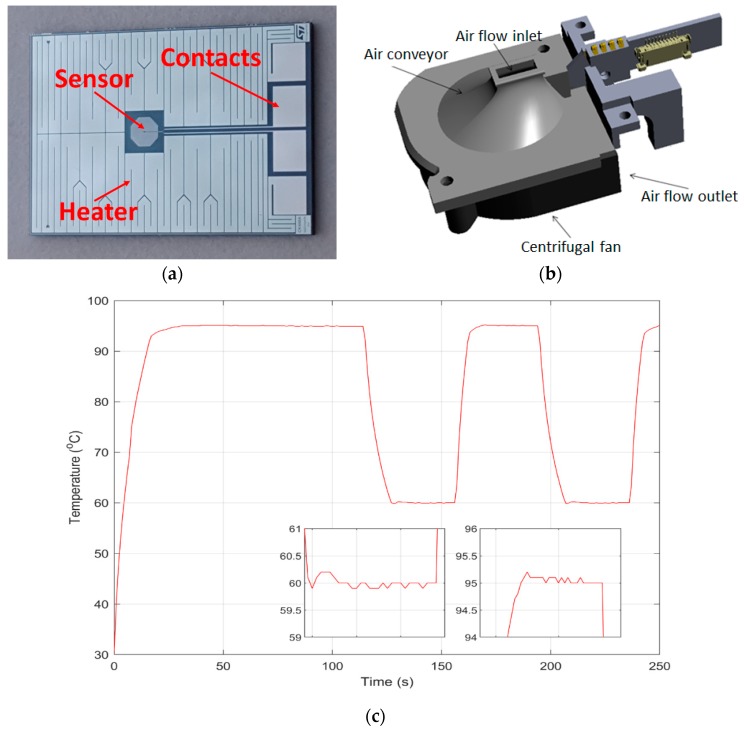
(**a**) Flipside of the silicon chip: the heater is a printed serpentine occupying most of the area. The temperature sensor is in the center of the heated area; (**b**) Partial view of the mechanics underneath the cartridge holder. A curved, air conveying surface directs the aspirated hot air to the centrifugal fan underneath; (**c**) Example of thermal curve as obtained from the onboard chip sensor. The initial ramp is visible, followed by the earliest cycles—a long-denaturation protocol, with equal denaturation and annealing/extension times was set for clarity.

The heater is part of the disposable LoC cartridge. In particular, it is a metal resistor made of aluminum (nominal value 20 Ω, power dissipated around 7 W at 12 V) printed on the back of the silicon chip (Figure 2a) which, thanks to the thermal conductivity of silicon, allows heat to distribute fast and uniformly to the wells on the other side. Two different sensors, one disposable—integrated in the cartridge—and one integrated in the instrument, measure the temperature. The disposable sensor is a small thermistor made of aluminum, printed on the flipside of the silicon die, near the heater (Figure 2a) and featuring ≈1 kΩ nominal value at room temperature and a temperature coefficient as large as 4.49384 × 10^−3^ °C^−1^. Although it provides fast and precise response to temperature variations, it does not directly measure the actual temperature inside the reaction chamber. Because of this and of the unavoidable spread in the value of the heating resistor—and sensor itself—each cartridge needs a preliminary calibration phase, during which the chip is slightly heated while the non-disposable sensor (featuring ±0.2 °C accuracy) measures the actual temperature. Calibration lasts three minutes and provides accurate and precise values for the subsequent temperature control phase, based on the chip-embedded resistive sensor, which, being in close proximity to the reaction mixture, measures temperature almost *in loco*.

Cooling is provided by a centrifugal fan (around 50 mm in diameter producing a 0.160 m^3^/min airflow at about 6500 rpm) located inside the main body of the instrument, under the cartridge. Hot air is aspirated away from the chip through a convex cavity, allowing fast and uniform chip cooling (Figure 2b).

Thanks to the low thermal inertia of the chip and good thermal conductivity of silicon, the thermal ramps are faster than in standard thermocyclers. In particular, the maximum temperature rates during heating and cooling are about +15 and −7 °C/s, respectively. The control accuracy is ±0.1 °C while the overall thermal accuracy is ±0.25 °C after calibration—comparable to that of the non-disposable sensor (see thermal curve and insets in Figure 2c). Moreover, the almost *in loco* temperature control, coupled with the small dimensions and low reagent volumes, enable shorter maintaining times in the thermal cycling steps.

### 2.4. Optical Read-Out

Optical detection relies on the fluorescent markers in common use in larger instruments. In fact, Q3-Plus V2 has been successfully tested on most of the fluorophores commonly used for qPCR detection (the tested fluorophores are listed in detail here below).

**Figure 3:**
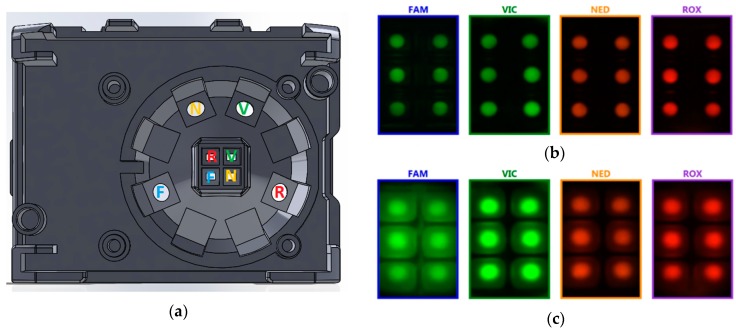
(**a**) Layout of the optical module, integrated in the upper lid of the instrument, evidencing the filtered LED sources (around) and the corresponding filtered CMOS sensors (in the center); (**b**) Fluorescent pictures from the four detection channels, with melted wax. The software determines the precise position of the circular regions of interest—i.e., the drops of reaction mixture plus sample—for accurate integration of the fluorescence signal; (**c**) Fluorescent pictures at low temperature: light scattering from solid-state wax outlines the area of the wells, compared with the reagents’ drops inside.

In its present version, the system handles up to four different detection channels, thus supporting multiplexing, i.e., amplifying and detecting more target sequences in the same well. For each channel, a suitable Light Emission Diode (LED) coupled with an excitation filter guarantees excitation. On the sensor side, each wavelength range is detected by a dedicated Complementary Metal-Oxide Semiconductor (CMOS) image sensor, in turn coupled with an emission filter. Figure 3a shows the layout of the optical module, integrated in the lid of the instrument, with the sources (LEDs + excitation filters) along the outer corona and the read-out units (CMOS image sensors + emission filters) in the central region—with diagonals of the active areas intersecting above the center of the cartridge underneath. Notice the antipodal positioning of paired sources/sensors, for better illumination uniformity.

For excitation in channel 1 (tested with FAM™/SYBR^®^ Green/EvaGreen^®^ dyes), a blue LED is used (wavelength: 465–485 nm). Two green LEDs, with dominant wavelength in the 520–530 nm interval, provide excitation for channel 2 (tested with VIC™/HEX™/TET™/JOE™/MAX dyes) and channel 3 (tested with NED™ and TAMRA dyes); an amber LED (wavelength: 590–595 nm) is for excitation in channel 4 (tested with ROX™ and Texas Red^®^ dyes). Each LED is coupled with a single band-pass excitation filter, tuned for optimal excitation of the detectable dyes in channel 1 (460–490 nm), 2 (520–540 nm), 3 (545–565 nm), and 4 (570–590 nm), respectively.

One CMOS image sensor (by STMicroelectronics) per channel reads out the luminous signal. The read-out filters coupled with the respective sensors are in turn tuned for optimal measurement of the dyes detectable in channel 1 (515–535 nm), 2 (545–565 nm), 3 (570–590 nm), and 4 (620–680 nm), respectively.

Activation of the LEDs occurs sequentially, one at a time, at each read-out step—in general during the low-temperature phase—and photographs of the fluorescent emission from the wells are concurrently acquired (Figure 3b). The fluorescence intensity in each well is obtained by integration on the regions of interest—the small round spots in the picture, coinciding with the areas of the qPCR reaction mixture drops. Notice that the photographs are taken in a molten wax condition, since in the solid state the wax would considerably scatter the fluorescent light, reducing the measurement accuracy (compare with Figure 3c, taken at ambient temperature).

From each typical fluorescence-vs.-time sigmoidal curve, a threshold cycle value is obtained as usual (C_q_, standing for “quantification cycle”, or C_T_, according to the traditional denomination) which relates to the initial target DNA/RNA concentration. It is defined as the fractional cycle number at which the fluorescence curve intersects an arbitrary threshold, set significantly above the background level and within the exponential portion of the curve, where the signal intensity is precisely correlated with the starting target DNA/RNA quantity.

### 2.5. Software

A control software (the “Suite”) integrates all the functionalities needed to run analyses with the Q3 system—creation of protocols, setup and launching of tests, real-time monitoring of their progress, and post analysis of generated data—in an environment similar to that of the standard thermocycler control software. The Suite is general-purpose and enables great versatility in executing the tests, while remaining easy to use. Standard qPCR protocols can be run, as well as isothermal protocols and melting/annealing analyses of the amplified products. Up to six Q3-Plus V2 instruments can be controlled from the same software session, with a random access approach.

With regard to data analysis, the software integrates the fitting of raw fluorescence data, calculation of the C_q_ value as well as melting temperature (T_m_, for melting analyses) of each curve, and the relative and absolute quantification of the starting quantities of target DNA/RNA sequences.

Once an assay has been optimized in all its aspects, a “closed version” of the software can also be developed, dedicated to that particular application, in which everything is pre-set—from thermal protocol to detection parameters, to data analysis and diagnostic interpretation. The operator in this case is not required—nor allowed—to change any parameter, which greatly reduces the risk of mistakes and makes the closed version even easier to use than the general-purpose one. Closed versions can run on PC as well as on smartphones/tablets, exploiting the Bluetooth^®^ connection capability of Q3-Plus V2.

## 3. Materials and Methods

### 3.1. Intra-Run Repeatability and Inter-Run Reproducibility: Comparison with a Gold Standard

All the protocols herein described were run on multiple ST Q3-Plus V2 instruments with 5 µL-cartridges and, in parallel, on a single CFX96™ Real-Time System instrument (Bio-Rad Laboratories, Hercules, CA, USA) with standard PCR plates. When comparing the performances between the two platforms, the same batch of qPCR reaction mixture was always used, as well as the same samples. The reaction volume was always 5 µL on both platforms.

*Protocol 1.* Four Q3 cartridges were loaded in parallel with four standard PCR plates (for CFX96 qPCR). Both Q3 cartridges and standard plates had six reaction wells each, loaded with the same qPCR duplex reaction mixture and the same sample, for a total reaction volume of 5 µL/well, comprising: 2.5 µL of TaqMan^®^ Fast Advanced Master Mix 2X (Thermo Fisher Scientific, Waltham, MA, USA), 1 µM each “X” primer (targeting a 67-base pair sequence on the human X chromosome; forward primer sequence: 5′-TGCACTGCGGCTTGTACAC-3′, reverse primer sequence: 5′-TCCACATTTCTAACTCCAGTTCACA-3′; Thermo Fisher Scientific), 300 nM “X” TaqMan^®^ MGB probe (FAM™-labelled hydrolysis probe, with sequence: 5′-FAM-ACAAGCTACTTGGTGGGAT-MGBNFQ-3′; Thermo Fisher Scientific), 1.3 µM each “Y” primer (targeting a 54-base pair sequence on the human Y chromosome; forward primer sequence: 5′-CGTCGGAAGGCGAAGATG-3′, reverse primer sequence: 5′-GGGATCTGCGGGAAGCA-3′; Thermo Fisher Scientific), 500 nM “Y” TaqMan^®^ MGB probe (VIC™-labelled hydrolysis probe, with sequence: 5′-VIC-TGCCGAAGAATTGCAG-MGBNFQ-3′; Thermo Fisher Scientific), and 2 µL of sample. The sample was in turn made of 3 × 10^4^ copies/reaction of a synthetic single-stranded DNA fragment (Sigma-Aldrich, Saint Louis, MO, USA), containing the sequence amplified by “X” primers, as well as 3 × 10^4^ copies/reaction of another synthetic single-stranded DNA fragment (Sigma-Aldrich), containing the sequence amplified by “Y” primers, both diluted in TE buffer 1×.

For Q3 qPCR, the thermal protocol was set as follows: initial denaturation at 95 °C for 120 s, followed by 40 cycles between 95 °C for 1 s and 58 °C for 20 s. The Q3-Plus V2 optical parameters were set as follows: exposure time 1/2 s, gain 14, LED power 5, for Channel 1; exposure time 1/2 s, gain 15, LED power 8, for Channel 2.

For CFX96 qPCR, the thermal protocol was: initial denaturation at 95 °C for 20 s, followed by 40 cycles between 95 °C for 3 s and 58 °C for 30 s.

*Protocol 2.* Four Q3 cartridges were loaded in parallel with four standard PCR plates (for CFX96 qPCR). Both Q3 cartridges and standard plates had six reaction wells each, loaded with the same qPCR triplex reaction mixture and the same sample, for a total reaction volume of 5 µL/well, comprising: 2.5 µL of TaqMan^®^ Fast Advanced Master Mix 2X, 600 nM Epstein-Barr Virus (EBV)-specific forward and reverse primer (Clonit S.r.l., Milan, Italy), 500 nM EBV hydrolysis probe (FAM™-labelled; Clonit) (EBV primers and probe are part of the CE-IVD marked “quanty EBV” assay, Clonit), 600 nM *Plasmodium ovale*-specific forward and reverse primer (Clonit), 500 nM *P. ovale* hydrolysis probe (VIC™-labelled; Clonit) (*P. ovale* primers and probe are part of the CE-IVD marked “Malaria Panel” assay, Clonit), 600 nM Hepatitis B Virus (HBV)-specific forward and reverse primer (Clonit), 500 nM HBV hydrolysis probe (ROX™-labelled; Clonit), and 2 µL of sample. The sample contained 6.6 × 10^4^ copies/reaction of each of three DNA plasmids (Clonit), each of them containing one of the three target sequences, all diluted in TE buffer 1×.

For Q3 qPCR, the thermal protocol was set as follows: initial denaturation at 95 °C for 120 s, followed by 40 cycles between 95 °C for 1 s and 60 °C for 20 s. The optical parameters for Q3-Plus V2 instrument were: same settings as in Protocol 1 for Channel 1 and 2; exposure time 1/2 s, gain 15, LED power 7, for Channel 4.

For CFX96 qPCR, the thermal protocol was set as follows: initial denaturation at 95 °C for 20 s, followed by 40 cycles between 95 °C for 3 s and 60 °C for 30 s.

*Protocol 3.* Three Q3 cartridges were loaded in parallel with three standard PCR plates (for CFX96 qPCR). Both Q3 cartridges and standard plates had six reaction wells each, loaded with the same Reverse Transcription (RT)-qPCR duplex reaction mixture and the same sample, for a total reaction volume of 5 µL/well, comprising: 1.25 µL of TaqMan^®^ Fast Virus 1-Step Master Mix 4X (Thermo Fisher Scientific), 0.25 µL of *LIG3* TaqMan^®^ Gene Expression Assay FAM™ for a final concentration of 900 nM forward and reverse primer and 250 nM probe (Assay ID: Hs00242692_m1, Thermo Fisher Scientific, a blend of two primers and a TaqMan^®^ MGB probe FAM™-labelled, specific for human *LIG3* mRNA), 0.25 µL of *GAPDH* TaqMan^®^ Gene Expression Assay VIC™ primer-limited for a final concentration of 150 nM forward and reverse primer and 250 nM probe (Assay ID: Hs99999905_m1, Thermo Fisher Scientific, a blend of two primers and a TaqMan^®^ MGB probe VIC™-labelled, specific for human *GAPDH* mRNA), 1.25 µL of ultrapure Milli-Q^®^ water, and 2 µL of sample. The sample consisted in human total RNA, extracted from oral squamous cell carcinoma tissue with the RNeasy Mini Kit (QIAGEN, Hilden, Germany) following the manufacturer’s instructions; after RNA quantification with NanoDrop™ 2000 (Thermo Fisher Scientific), the sample was diluted with TE buffer 1× to a concentration of 2.5 ng/µL. Thus, the sample for qPCR contained 5 ng/reaction of human total RNA.

On Q3, the thermal protocol was set as follows: reverse transcription at 55 °C for 360 s, followed by initial denaturation at 95 °C for 30 s, and then 40 cycles between 95 °C for 5 s and 60 °C for 30 s. The optical parameters were set as in Protocol 1.

On CFX96, the thermal protocol was the same as on Q3.

### 3.2. Linearity and Sensitivity

All the protocols herein described were run on ST Q3-Plus V2 instruments with 5 µL-cartridges.

*Protocol 4*. Three µL of the same qPCR duplex reaction mixture as in Protocol 1 were loaded in each well of the tested Q3 cartridges, containing in brief: 2.5 µL of TaqMan^®^ Fast Advanced Master Mix 2X, 1 µM each “X” primer, 300 nM “X” hydrolysis probe FAM™-labelled, 1.3 µM each “Y” primer, and 500 nM “Y” hydrolysis probe VIC™-labelled (all primers’ and probes’ sequences are detailed hereinabove in Protocol 1). Two µL of a different sample were then loaded in each well, so that each well had a different concentration (in a 10-fold dilution series) of two synthetic single-stranded DNA fragments containing the sequence amplified by “X” and “Y” primers, respectively (same fragments as in Protocol 1), diluted in TE buffer 1×, for a total reaction volume of 5 µL/well. The first well had 3 × 10^7^ copies/reaction of each of the two DNA templates, down to the sixth well that contained 3 × 10^2^ copies/reaction of each of the two DNA templates.

The thermal protocol was set as in Protocol 1, except that 45 cycles were run. The Q3-Plus V2 optical parameters were set as in Protocol 1.

*Protocol 5*. Three µL of a qPCR singleplex reaction mixture were loaded in each well of the tested Q3 cartridges, containing: 2.5 µL of TaqMan^®^ Fast Advanced Master Mix 2X, 600 nM each EBV primer (Clonit), and 500 nM EBV hydrolysis probe (FAM™-labelled; Clonit). Two µL of a different sample were then loaded in each well, so that each of them had a different concentration (in a 1.3-fold dilution series) of a DNA plasmid (Clonit), diluted in TE buffer 1×, for a total reaction volume of 5 µL/well. The plasmid contained the sequence amplified by EBV primers. The first well contained 2 × 10^5^ copies/reaction of plasmid, down to the sixth well that contained 5.4 × 10^4^ copies/reaction of plasmid.

The thermal protocol was set as follows on Q3-Plus V2: initial denaturation at 95 °C for 120 s, followed by 40 cycles between 95 °C for 1 s and 61 °C for 20 s. The optical parameters were set as follows for Channel 1: exposure time 1/2 s, gain 13, LED power 5.

*Protocol 6*. Three µL of a qPCR singleplex reaction mixture were loaded in each well of the tested Q3 cartridges, containing: 2.5 µL of TaqMan^®^ Fast Advanced Master Mix 2X, 800 nM each “X” primer, and 300 nM “X” hydrolysis probe FAM™-labelled (primers’ and probe’s sequences are detailed hereinabove in Protocol 1). Two µL of sample were then added to each well, for a total reaction volume of 5 µL/well. In detail, the sample was human male genomic DNA, extracted from 200 µL of whole blood with the ReliaPrep™ 96 gDNA Miniprep HT System kit (Promega Corporation, Madison, WI, USA) according to the manufacturer’s instructions. The concentration and purity of extracted DNA were assessed with NanoDrop™ 2000 (Thermo Fisher Scientific) and with qPCR on CFX96, by comparing the obtained C_q_ value with that of a commercial genomic DNA sample (TaqMan™ Control Genomic DNA human male, Thermo Fisher Scientific). The extracted DNA was then serially diluted 1:10 with TE buffer 1×, up to 1:10^4^ dilution factor. In each Q3 tested cartridge, then, five wells were loaded with 2 µL of the undiluted sample (3 × 10^4^ genome copies/reaction) and of the four serial dilutions (down to 3 genome copies/reaction), respectively; the sixth well was a no-template control (NTC), in which 2 µL of ultrapure Milli-Q^®^ water took the place of the sample.

The thermal protocol was set as in Protocol 1, except that 45 cycles were run. The optical parameters for Channel 1 were the same as in Protocol 1.

## 4. Results

### 4.1. Intra-Run Repeatability and Inter-Run Reproducibility: Comparison with a Gold Standard

These tests tackle the uniformity of results across the six wells, either on the same or on different cartridges. Assuming accurate loading with the same quantities of reagents and sample, the reliability across the wells typically depends on uniform temperature distribution and optical read-out characteristics, as well as on the qPCR-compatibility of the cartridge materials contacting the reagents. To test this aspect, in a first experimental setup, equal concentrations of two different target DNA sequences were loaded in the six wells of each of four cartridges, together with the same reaction mixture for the two targets. Then, qPCR analyses were run on four different Q3-Plus V2 instrument units, according to Protocol 1 detailed in Materials and Methods. As a reference, four qPCR runs were executed in parallel on a single CFX96™ Real-Time System instrument (Bio-Rad Laboratories), each involving six wells loaded with the same reaction mixture and sample as in the four Q3 cartridges.

**Figure 4:**
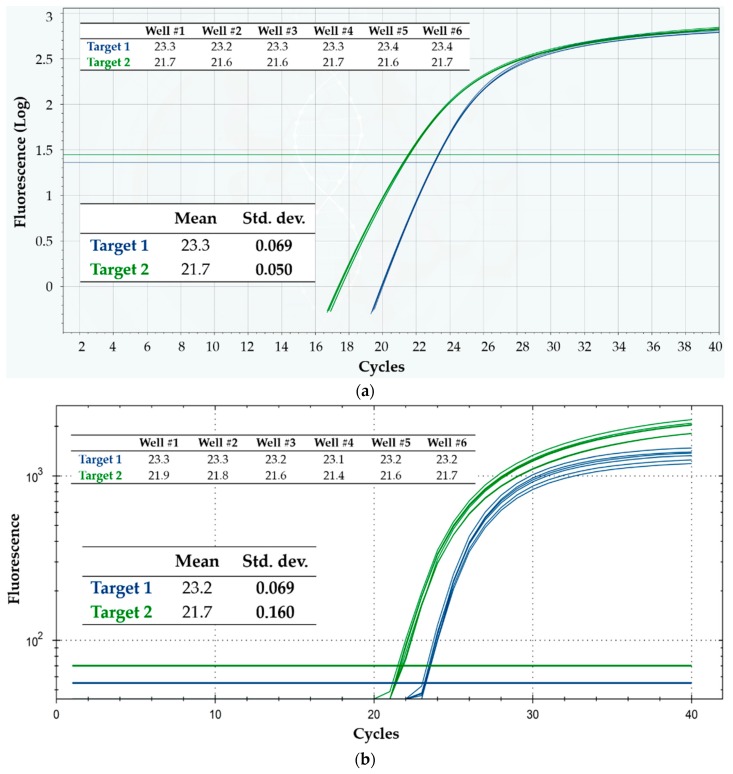
Intra-run repeatability on the detection of two DNA targets, using Protocol 1. Shown are the fluorescence vs. cycle qPCR curves; blue curves refer to target 1, while green ones refer to target 2. The inset tables display the corresponding C_q_ values, as well as their mean and standard deviation (Std. dev.). (**a**) Q3-Plus V2 results from a cartridge; (**b**) CFX96 results from a run.

Figure 4a shows the results obtained on a Q3 cartridge, indicating very high repeatability of the curves for both targets, especially around threshold and in the exponential zone. The standard deviations of C_q_, reported in the inset table of the figure were less than 0.3% of the mean value for both targets, confirming high repeatability and, so, precise target DNA quantification. In fact, assuming reaction efficiency close to 100% and normally distributed C_q_ values—both assumptions being valid in most cases—the “worse” standard deviation value (0.069 for target 1) means that, in a run executed under these experimental conditions, the Q3 system is able to correctly quantify (and thus distinguish) in 99.7% of cases different samples having only a 1.3-fold difference in target quantity. Such resolution in quantifying is far more than what is typically needed in most diagnostic applications, and is usually considered as the resolution limit of qPCR [14]. If different samples had a 2-fold ratio in target quantity, the probability of correctly discriminating them would become higher than 99.99%, at equal standard deviation.

Analogously, and for the sake of comparison, Figure 4b displays the results obtained on a Bio-Rad CFX96 run, while Table 1 summarizes the overall results from both the four Q3 cartridges and the four CFX96 runs. The C_q_ value from each well of the Q3 cartridges was definitely repeatable intra-cartridge, for both targets and in all the four tested cartridges. Notice also that, remarkably, target 2 showed better intra-run repeatability on Q3 than on the reference instrument.

It is worth noting that C_q_ values generated from different runs are notoriously subject to inherent inter-run variation, hence reporting inter-run C_q_ variation is not typically appropriate [15]. Nevertheless, the Q3 system also showed a very high inter-run reproducibility across all the four runs—much higher than on CFX96—as evidenced in Table 1. The standard deviations of C_q_ in CFX96 were 1.7- and 3.9-fold larger than the corresponding in Q3, respectively for targets 1 and 2. This demonstrates the consistency of different cartridges and Q3-Plus V2 instrument units, and potentially paves the way to reliable quantification in unknown samples without the need for standard samples for calibration on board the cartridge.

The denaturation and annealing/extension times were set shorter on Q3 than on CFX96—1 vs. 3 s, and 20 vs. 30 s, respectively—to test both platforms in the best respective working conditions. For CFX96, specific instructions by the manufacturer of the used master mix were followed [16]. For Q3, the timings suggested by the same manufacturer for benchtop thermocyclers with fast cycling mode were used [17] (p. 14), to exploit the peculiar characteristics of Q3 system (see Section 2.3). A faster protocol is potentially more challenging, thus, on absolute terms, the Q3 could have been slightly disfavored—and yet, its performances were comparable to those of CFX96 or slightly better. On the other hand, the wax pre-loaded inside the Q3 cartridge wells required an increased initial denaturation time—120 vs. 20 s on CFX96—for complete melting. Overall, thanks also to faster thermal ramps, the Q3 ran its protocol in around 40 min, compared to around 1 h required by CFX96 for its own.

In order to further test the Q3 system in more challenging conditions, the same kind of experiment was also run with a triplex reaction mixture—that is, analyzing three different DNA targets in each cartridge well, by exploiting the multi-channel optics of the Q3-Plus V2 instrument—using different targets than in Protocol 1. High multiplexing further stresses the system, since optical uniformity has to be guaranteed in each read-out channel, and is more challenging on the chemical side as well. Six wells in Q3 cartridges, and six in standard plates were then loaded in each of four runs, on both platforms; the qPCR protocol was as described in Protocol 2 in Materials and Methods.

**Figure 5:**
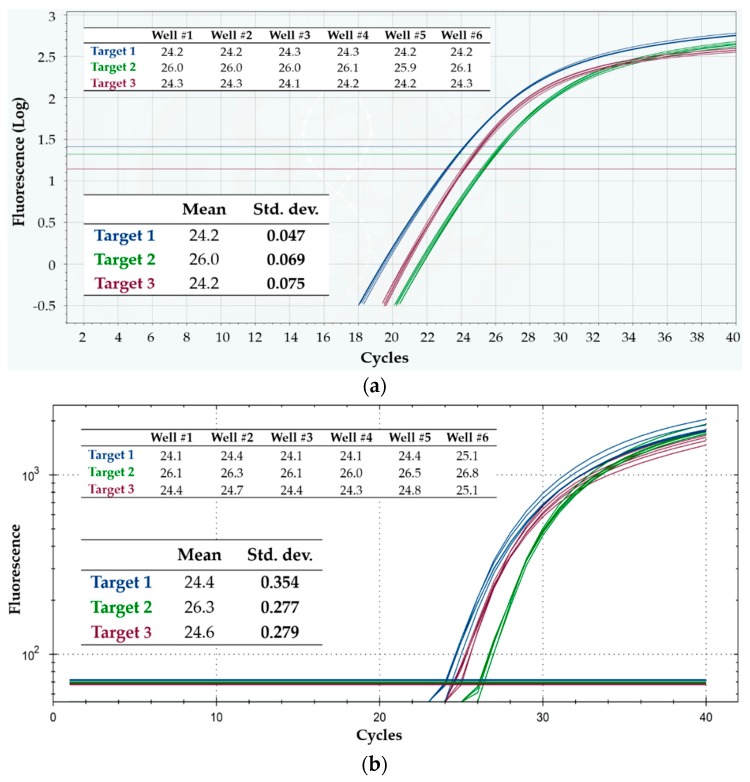
Intra-run repeatability on the detection of three DNA targets, using Protocol 2. Shown are the fluorescence vs. cycle qPCR curves; blue curves refer to target 1, green curves refer to target 2, and purple curves refer to target 3. The inset tables display the corresponding C_q_ values, as well as their mean and standard deviation (Std. dev.). (**a**) Q3-Plus V2 results from a cartridge; (**b**) CFX96 results from a run.

Figure 5a,b show the results from a Q3 cartridge and from a CFX96 run, respectively—in particular, the qPCR curves, C_q_ values and their standard deviations—while Table 2 summarizes all the results from both platforms. Again, the intra-run repeatability proved very high in Q3 for all of the three targets/optical channels—and much better than in CFX96 (standard deviations in the latter about 3- to more than 4.5-fold larger than in the former). Moreover, as shown in Table 2, the inter-run reproducibility was again high in Q3, and lower in CFX96. Hence, the Q3 system demonstrated to be very precise in C_q_ calculation even when challenged with a triplex reaction mixture.

Last, the same type of test was run with a duplex assay where the targets were two different RNA sequences, co-amplified and detected in each well. Analyzing RNA instead of DNA with the so-called Reverse Transcription qPCR (RT-qPCR) is more challenging, for two reasons. First, RNA is more labile than DNA; thus, were the cartridge materials not perfectly “RT-qPCR-compatible”, RNA amplification might be impaired/inhibited unpredictably, and variably across the wells. Second, when RNA is the target, an additional reaction step is needed at the beginning—the reverse transcription—typically occurring at around 50–55 °C. This step might have different susceptibility to a possible temperature non-uniformity across the wells. For these tests, the same RNA sample was loaded in all the six wells of three cartridges, together with the same duplex reaction mixture, and RT-qPCR was run according to Protocol 3, as detailed in Materials and Methods. In parallel, three runs were executed on a CFX96 instrument as a reference, each run consisting of six loaded wells.

**Figure 6:**
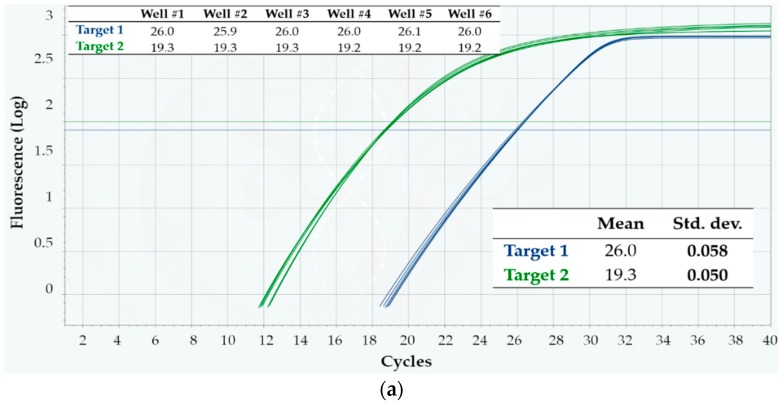

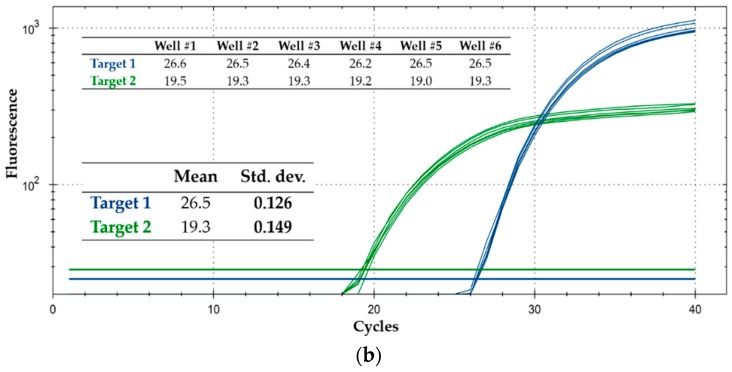
Intra-run repeatability on the detection of two RNA targets, using Protocol 3. Shown are the fluorescence vs. cycle qPCR curves; blue curves refer to target 1, while green ones refer to target 2. The inset tables display the corresponding C_q_ values, as well as their mean and standard deviation (Std. dev.). (**a**) Q3-Plus V2 results from a cartridge; (**b**) CFX96 results from a run.

Figure 6a,b show the RT-qPCR curves, as well as the average and standard deviations of C_q_ values from a Q3 cartridge and a CFX96 run, respectively, while Table 3 contains a summary from both platforms. Once more, the intra-cartridge repeatability was found higher in Q3, for both targets—and higher than in CFX96 (by about a 2.5 factor). The same held for the inter-run reproducibility, with standard deviations about 2.8-fold larger in CFX96 than in Q3. Thus, precise quantification of target RNA sequences is also possible with the Q3 system, even when multiplexing.

### 4.2. Linearity and Sensitivity

The second kind of validation tests herein presented challenges two other key parameters of qPCR: linearity, that is, the correspondence of C_q_ value to the initial number of target DNA/RNA copies, and analytical sensitivity, which is the minimum number of target copies that can be reliably detected. Both parameters chiefly depend again on the uniformity of the optical read-out and temperature across the wells, as well as on the qPCR-compatibility of the cartridge materials contacting the reagents and on each qPCR assay itself. High sensitivity also requires the instrument’s optics to be adequately sensitive.

With regard to linearity, due to the exponential regime of DNA amplification in qPCR, the C_q_ value is expected to decrease linearly vs. increasing logarithmic initial concentration of target sequence. To check the prediction, some cartridges were loaded in a way that each well contained a different concentration (10-fold dilution series) of two target DNA sequences, thus spanning a 6-log_10_ dynamic range of input template amount. All the wells were also loaded with the same duplex reaction mixture for those two targets—which were the same as in Protocol 1—and qPCR analysis was then run on Q3-Plus V2 according to Protocol 4, as described in Materials and Methods.

**Figure 7:**
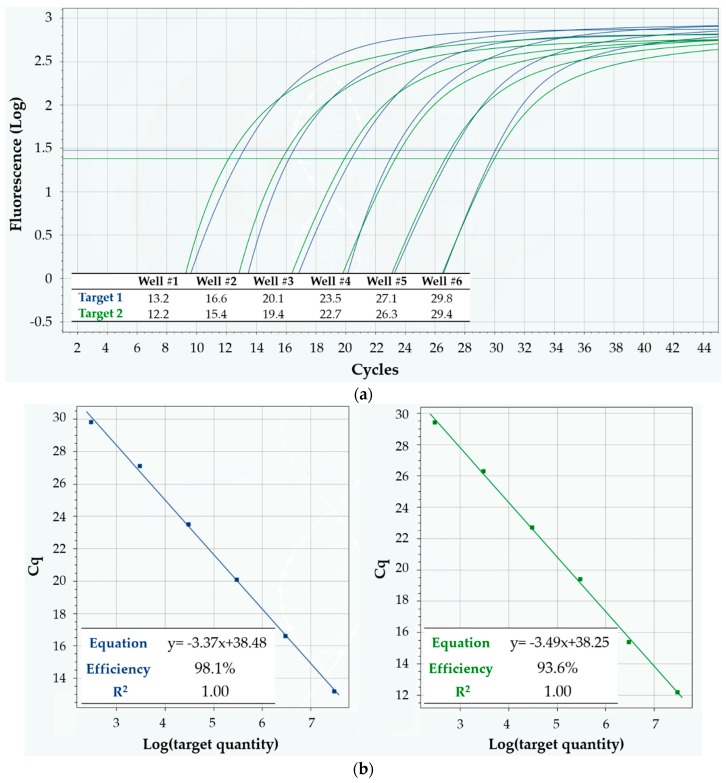
Linearity of Q3-Plus V2 in multiplex detection of two DNA targets in a 10-fold dilution series, using Protocol 4. (**a**) Fluorescence vs. cycle qPCR curves. Blue curves refer to target 1, while green ones refer to target 2. The inset table displays the corresponding C_q_ values; (**b**) Standard curves for absolute quantification of target quantity. The inset tables show the equation of the linear fit, as well as its R^2^ value and the mean reaction efficiency for target 1 (left, in blue) and target 2 (right, in green).

Typical qPCR curves obtained on a cartridge are shown in Figure 7a. The corresponding linearity results are summarized in Figure 7b: shown are two classic standard curves for absolute quantification, one for each target, obtained from the same cartridge. The data are in excellent agreement with the predictions, with the linear model scoring R^2^ = 1 for both targets, which amounts to perfect linearity of C_q_ values and correspondingly reliable absolute quantification of target sequences within the allowed precision. Moreover, the mean reaction efficiency (calculated from the slope of the linear fit, according to a standard formula) was 98.1% for target 1, and 93.6% for target 2—definitely lying inside the very good efficiency range (90–110%, when efficiency is calculated with this method [18]).

In a second set of tests, the linearity of Q3 system was again assessed, evaluating its discrimination power when analyzing samples in which the target quantity differed by only a 1.3-fold factor. To this scope, some cartridges were loaded in a way that each well contained a different concentration (1.3-fold dilution series) of a target DNA sequence. All the wells were also loaded with the same reaction mixture for that target, and qPCR analysis was run on Q3-Plus V2, according to Protocol 5 described in Materials and Methods.

**Figure 8:**
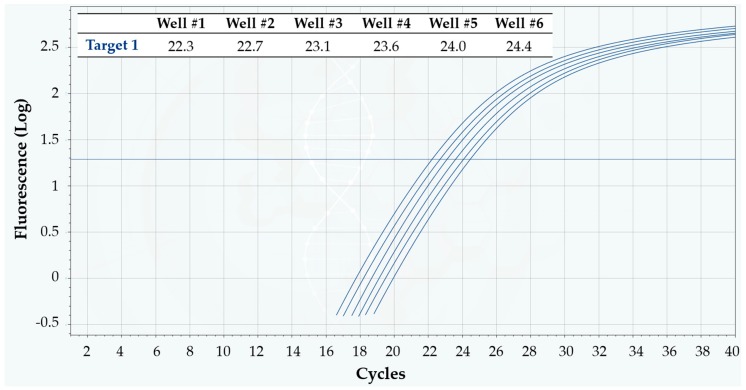
Linearity of Q3-Plus V2 in detecting a DNA target in a 1.3-fold dilution series, using Protocol 5. The fluorescence vs. cycle qPCR curves appear clearly separated around the threshold, meaning that even such small differences in target quantity can be reliably detected. The inset table displays the corresponding C_q_ values. The mean difference ΔC_q_ between subsequent dilutions is 0.4—as expected for a reaction efficiency around 100%.

Figure 8 illustrates the typical results obtained on a cartridge in this test session. The C_q_ values in the inset table, as well as the separation of curves around the threshold, clearly demonstrate that Q3 was able to achieve efficient target quantity discrimination at 1.3-fold differences—value which is typically considered as the limit of qPCR resolution [14]. The mean ΔC_q_ value between one dilution and the following was 0.4, which is exactly the expected value for 1.3-fold dilutions, when the reaction efficiency is around 100%.

In a third set of experiments, besides linearity, the analytical sensitivity of Q3 system was also assessed. To this scope, some cartridges were loaded in a way that each of the first five wells contained a different quantity (10-fold dilution series) of a human genomic DNA sample, down to only 3 target copies in the fifth well. The sixth well was instead a no-template control (NTC), with no DNA inside, which must yield a negative result in order to exclude possible contaminations. All the wells were then loaded with the same reaction mixture for a human target DNA, and qPCR analysis was run on Q3-Plus V2 according to Protocol 6, as described in Materials and Methods.

**Figure 9:**
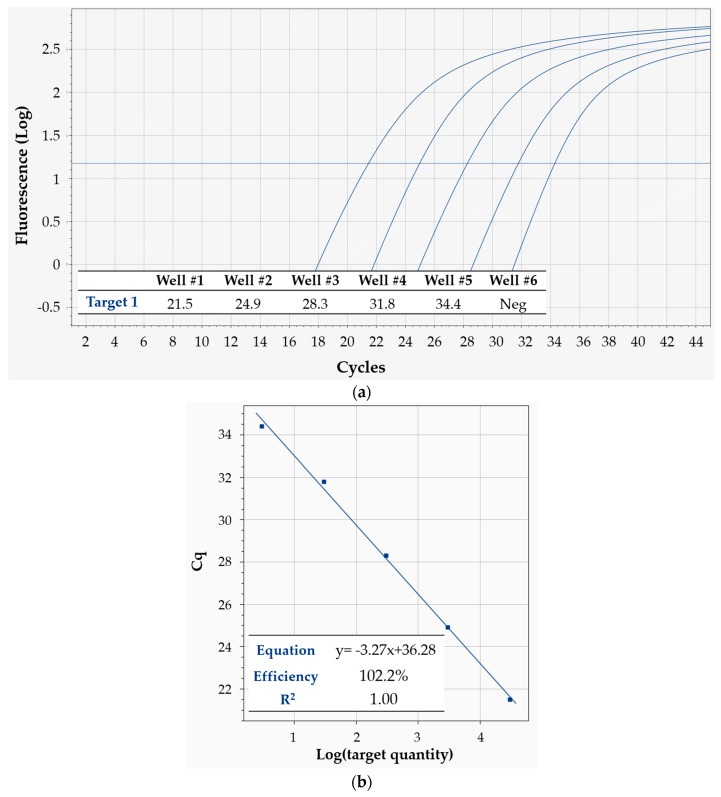
Linearity and sensitivity of Q3-Plus V2 in detecting a DNA target in a 10-fold dilution series, using Protocol 6. (**a**) Fluorescence vs. cycle qPCR curves. The inset table displays the corresponding C_q_ values. The sixth well featured a no-template control—which yielded a negative result, as expected; (**b**) Standard curve for absolute quantification of target quantity. The inset table shows the equation of the linear fit, as well as its R^2^ value and the mean reaction efficiency.

Figure 9a shows the qPCR curves from one cartridge, while Figure 9b displays the corresponding standard curve, its equation, the calculated efficiency and R^2^. Again, the obtained C_q_ values perfectly agreed with the linear fit, as seen from R^2^ = 1. The calculated mean reaction efficiency was 102.2%—comfortably lying inside the range that is considered optimal when efficiency is calculated with this standard method, i.e., 90–110% [18]—indicating perfect efficiency. As to sensitivity, the most diluted sample contained only 3 target DNA copies/reaction nominally—and yet, it was clearly detectable. Very importantly, the NTCs of all cartridges resulted negative as expected—ruling out any possible contamination artefacts in the detection of low-concentration samples, and confirming the high sensitivity of Q3-Plus V2. This result is even more remarkable if one considers that it was obtained on human genomic DNA, purified from whole blood—meaning that extreme sensitivity can also be achieved on real specimens. It is also worth pointing out that sensitivity not only depends on the performance of the system, but also on the specific qPCR reaction assay used for the analysis.

## 5. Conclusions

Porting qPCR tests onto a reliable on-chip system apt to commercial exploitation requires accurate engineering work, especially because of the required precision in the control of thermal parameters and read-out of the output signals. The Q3-Plus V2 system here described reaches the goal and it does, remarkably, on seamlessly ported commercial tests. In this sense, to our knowledge, Q3-Plus V2 is one of the most mature, general-purpose on-chip systems for qPCR being currently available. We have demonstrated that its performances are adequate, as well as completely comparable to—if not sometimes even better than—those of a gold standard, commercial benchtop qPCR instrument.

## Figures and Tables

**Table 1:** Overall results from the intra-run repeatability and inter-run reproducibility tests on the detection of two DNA targets, using Protocol 1. The second and third columns display the mean and standard deviation (Std. Dev.) of C_q_ values from each of the four runs executed in four Q3-Plus V2 (Q3) instruments and in one CFX96 instrument, for both targets. The fourth column (Mean Std. Dev.) shows the average of the four intra-run standard deviation values for both targets in each platform—Q3 and CFX96. The fifth and sixth columns show the inter-run—that is, the overall—mean and standard deviation of C_q_ values for both targets in each platform.

	Intra-Run			Inter-Run	
	Mean	Std. Dev.	Mean Std. Dev.	Mean	Std. Dev.
Q3, Target 1	23.2	0.090	0.074	23.2	0.094
	23.3	0.069			
	23.2	0.069			
	23.2	0.069			
CFX96, Target 1	23.2	0.069	0.065	23.4	0.163
	23.6	0.047			
	23.4	0.075			
	23.4	0.069			
Q3, Target 2	21.6	0.047	0.060	21.7	0.087
	21.7	0.050			
	21.8	0.069			
	21.7	0.075			
CFX96, Target 2	21.7	0.160	0.144	22.2	0.346
	22.5	0.134			
	22.1	0.149			
	22.3	0.134			

**Table 2:** Overall results from the intra-run repeatability and inter-run reproducibility tests on the detection of three DNA targets, using Protocol 2. The second and third columns display the mean and standard deviation (Std. Dev.) of C_q_ values from each of the four runs executed in four Q3-Plus V2 (Q3) instruments and in one CFX96 instrument, for the three targets. The fourth column (Mean Std. Dev.) shows the average of the four intra-run standard deviation values for the three targets in each platform—Q3 and CFX96. The fifth and sixth columns show the inter-run—that is, the overall—mean and standard deviation of C_q_ values for the three targets in each platform.

	Intra-Run			Inter-Run	
	Mean	Std. Dev.	Mean Std. Dev.	Mean	Std. Dev.
Q3, Target 1	24.1	0.075	0.069	24.1	0.143
	24.2	0.047			
	24.0	0.080			
	23.9	0.075			
CFX96, Target 1	24.4	0.354	0.322	24.5	0.369
	24.3	0.315			
	24.7	0.309			
	24.5	0.309			
Q3, Target 2	25.9	0.047	0.073	26.0	0.096
	26.0	0.069			
	26.0	0.075			
	26.1	0.100			
CFX96, Target 2	26.3	0.277	0.260	26.5	0.300
	26.4	0.267			
	26.7	0.219			
	26.6	0.275			
Q3, Target 3	24.2	0.111	0.109	24.2	0.127
	24.2	0.075			
	24.1	0.115			
	24.1	0.137			
CFX96, Target 3	24.6	0.279	0.315	24.7	0.324
	24.8	0.350			
	24.5	0.345			
	24.6	0.292			

**Table 3:** Overall results from the intra-run repeatability and inter-run reproducibility tests on the detection of two RNA targets, using Protocol 3. The second and third columns display the mean and standard deviation (Std. Dev.) of C_q_ values from each of the three runs executed in three Q3-Plus V2 (Q3) instruments and in one CFX96 instrument, for both targets. The fourth column (Mean Std. Dev.) shows the average of the three intra-run standard deviation values for both targets in each platform—Q3 and CFX96. The fifth and sixth columns show the inter-run—that is, the overall—mean and standard deviation of C_q_ values for both targets in each platform.

	Intra-Run			Inter-Run	
	Mean	Std. Dev.	Mean Std. Dev.	Mean	Std. Dev.
Q3, Target 1	26.0	0.058	0.063	26.0	0.076
	26.0	0.075			
	26.1	0.058			
CFX96, Target 1	26.6	0.273	0.158	26.5	0.207
	26.5	0.126			
	26.4	0.075			
Q3, Target 2	19.3	0.050	0.069	19.2	0.073
	19.2	0.090			
	19.2	0.069			
CFX96, Target 2	19.4	0.223	0.192	19.3	0.217
	19.3	0.149			
	19.1	0.205			
